# Supplementary material for: Chromothripsis during telomere crisis is independent of NHEJ, and consistent with a replicative origin
Source: Genome Res. 2019 May;29(5):737–49. doi: 10.1101/gr.240705.118 (PMC6499312; doi:10.1101/gr.240705.118)
Supplement: Supplemental Material [file supp_gr.240705.118_Supplemental_file_1.zip › contigs/annotated_contigs/DB115/contig.2.DB115_length_503_mean_cov_7.45526838966.docx]

**DB115_length_503_mean_cov_7.45526838966**

```
CAGAAATATTTGACTCCTTCCTGCCCCTACCTCTAGGCACTGACTCATTTTATTAATCTCATTAAATTCAGGAAGCTTTTGGATCATCT
 >chr6:103449683-103449892 - E=3e-114 p=4e-02
TCCAAACTAAATTAATAAAAAACTTAATTCTTCTCAGATTTAAGAGATCTCTCATGCAAGGAACTATATAATTTCCTGCTGTGCTTTTG

TAGAGCATTTGACCAATATTAGTATGATATT|AGTATGATA|TTGTTTAATAACATTTCAAATTTGTTAGTTGGCATCCCCATGGACAA
                                         >chr6:103270945-103271200 - E=7e-133
AAAGTTGATATTATAATATTATCCTAGTTAATCACCATAACTCCATCAAACATTATGTAGACTATAACAGTATACTAGGCAAATCCTGT

GCCTCTATGCAAGAGACATAAAGATATTTTCCCATTTTGAAGAAATTATATAATAAGAAAAATAATGGGAAGGAACAGTATTAAGCAAC

AGCTGAACATTGCTGACAAGGCCAGACAAG|AAAAGACAGTAGGGGAAAAAAGCAAAAAAA
```
